# Salivary bacterial community profile in normal‐weight and obese adolescent patients prior to orthodontic treatment with fixed appliances

**DOI:** 10.1111/ocr.12571

**Published:** 2022-05-01

**Authors:** Shima H. Sharara, Leanne M. Cleaver, Hayder Saloom, Guy H. Carpenter, Martyn T. Cobourne

**Affiliations:** ^1^ Centre for Craniofacial Development and Regeneration Department of Orthodontics Faculty of Dentistry, Oral & Craniofacial Sciences King's College London London UK; ^2^ Centre for Host‐Microbiome Interactions Department of Mucosal and Salivary Biology Faculty of Dentistry, Oral and Craniofacial Sciences King's College London London UK; ^3^ Orthodontic Department College of Dentistry University of Baghdad Baghdad Iraq

**Keywords:** adolescents, obesity, orthodontics, salivary microbiome

## Abstract

**Objectives:**

The aim of this study was to compare the intra‐oral bacterial profile of normal‐weight and obese adolescents prior to orthodontic treatment with fixed appliances.

**Materials and methods:**

Nineteen adolescent patients were recruited into two groups based upon body mass index (BMI) and classified as normal‐weight or obese. Unstimulated whole mouth saliva was obtained for 5 minutes. Bacterial DNA extraction was performed from saliva, and 16S rRNA gene sequencing of the V1‐2 variable regions was undertaken followed by analysis using the mothur pipeline.

**Results:**

Saliva from a total of 19 adolescent patients with mean (SD) age 15.6 (1.8) years were divided into 10 normal‐weight with mean BMI of 19.4 (2.2) kg/m^2^ and 9 obese with mean BMI of 30.2 (3.5) kg/m^2^. A total of 156 783 sequences were obtained from the 19 samples with no significant differences in richness or diversity between sample groups by obesity status or gender (AMOVA). The bacterial community in both groups was dominated by bacterial genera characteristic of the human mouth, which included *Streptococcus, Porphyromonas, Veillonella, Gemella, Prevotella, Fusobacterium* and *Rothia*.

**Conclusion:**

There were no differences in alpha or beta diversity of oral bacterial communities between normal‐weight and obese orthodontic patients. Obese adolescents attending for orthodontic treatment had a similar microflora to their normal‐weight counterparts.

## 1 INTRODUCTION

Obesity is a global health concern in both child and adult populations. In the United Kingdom alone, the prevalence of obesity has risen ten‐fold over the past four decades in both populations.^1^ Obesity causes a status of chronic subclinical inflammation in the human body, where adipose tissue can act as an endocrine organ, secreting inflammatory proteins or adipokines that act as both pro‐or anti‐inflammatory mediators.^2^ We have previously demonstrated elevated levels of the pro‐inflammatory adipokines leptin and resistin, the inflammatory marker myeloperoxidase and cytokine receptor for nuclear factor kappa‐B ligand (NFkB) in the gingival crevicular fluid (GCF) of obese orthodontic patients compared to normal‐weight, suggestive of a potentially altered inflammatory mechanism in the periodontal tissues of these individuals. In addition, significantly increased rates of tooth movement from baseline to the completion of alignment phase were identified amongst obese patients compared to normal‐weight, thus presenting potential short‐ and long‐term clinical implications.^3^ Obesity has also been strongly associated with an increased risk of caries, gingivitis and periodontitis in a variety of population groups.^4^, ^5^ A positive relationship has been shown between disturbances in the gut microbiome and an increased risk of obesity^6^; however, whilst differences in the salivary microbiota have been reported in obese adults,^7^, ^8^ other investigations have found no overall difference in the diversity of salivary microbiota in obese children.^9^

Oral and systemic conditions affecting an individual can manifest in the salivary microbiota.^10^, ^11^ A number of systemic conditions have been associated with alteration in oral microbial composition including polycystic ovary syndrome, hepatic encephalopathy and pancreatic cancer.^12^, ^13^, ^14^ Common oral conditions such as caries and periodontitis are associated with changes in oral bacterial composition, with a shift to aciduric species such as *Streptococcus mutans* and lactobacilli, in addition to *Bifidobacterium, Propionibacterium* and *Scardovia* in caries and *Porphyromonas gingivalis, Tannerella forsythia, Treponema denticola, Anaeroglobus geminatus, Eubacterium saphenum, Filifactor alocis, Porphyromonas endodontalis* and unnamed *Bacteroidetes* and *Fretibacterium* phylotypes in periodontitis.^10^, ^11^ Whilst increased bacterial richness in the salivary microbiome, more specifically *Prevotella* and *Viellonella*, is significantly associated with poorer oral health and reflected amongst patients with increased BMI and old age,^15^ other studies have reported no association between BMI and change in salivary microbial array^9^, ^13^, ^16^, ^17^ or low bacterial diversity.^18^ This is consistent with studies investigating the gut microbiota of obese adult and adolescent individuals, which have found low microbial diversity with lower *Bacteroidete*s and more *Firmicutes* compared to normal‐weight.^19^

There are clear associations between the wearing of orthodontic appliances and oral health, particularly in terms of enamel demineralization, caries and periodontal inflammation.^20^ Several studies have reported changes in the salivary microbiome amongst patients receiving orthodontic treatment towards periodontal pathogens, with increases in Gram‐positive bacteria from pre‐treatment to 1 year^20^, ^21^, ^22^ and in gram‐negative anaerobic bacteria,^21^, ^22^ which gradually returned from 3 months to 1 year post‐treatment.^21^ However, prospective data exist to suggest that microbial changes occur towards the end of orthodontic treatment, and if good oral hygiene is maintained, orthodontic treatment does not adversely affect oral health.^23^

There is emerging evidence reporting the relationship between the salivary microbiota and obesity, as well as the salivary bacterial community and orthodontics. To date, no studies have examined the salivary microbiome amongst obese adolescents undergoing orthodontic treatment and whether any differences might represent an increased risk for orthodontic iatrogenic effects such as periodontal disease. In this study, the salivary bacterial community in normal‐weight and obese subjects were analysed at baseline before the placement of fixed appliances.

## 2 MATERIALS AND METHODS

### 2.1 Study design

This cohort study compared the effects of obesity on the intra‐oral microbiological flora of adolescent orthodontic patients prior to the placement of fixed appliances. We report and present data according to STROBE (Strengthening the Reporting of Observational Studies in Epidemiology).^24^

### 2.2 Setting

Participants were recruited from the Department of Orthodontics at Guy's and St Thomas' NHS Foundation Trust in the Faculty of Dentistry, Oral & Craniofacial Sciences at King's College London between January 2015 and January 2016.

### 2.3 Participants

The inclusion criteria for participation in this study were: (1) age 12‐18 years at baseline; (2) non‐smoker; (3) no medical contraindications or regular medication (including antibiotic therapy) in the previous 6 months; and (4) normal‐weight (BMI‐centile 2‐91 kg/m^2^) or obese (BMI‐centile >98 kg/m^2^) classification, with underweight (BMI‐centile <2 kg/m^2^) and overweight (BMI‐centile 91‐98 kg/m^2^) subjects excluded. Once included, participants were assessed at baseline before undergoing fixed appliance orthodontic treatment. Sample size was based on data from a previous study investigating the microbial profile in saliva samples from patients diagnosed with periodontitis.^25^

### 2.4 Variables

Baseline data were collected as previously described.^3^ Briefly, unstimulated whole mouth saliva (uWMS) was obtained from subjects spitting into a plastic collection tube for 5 minutes and the uWMS rate calculated as millilitre per minute (mL/min). Periodontal health was measured clinically using established validated plaque and gingival indices.^26^, ^27^ Subject body weight was measured to the nearest 0.1 kilogram (kg) using a calibrated scale and height measured to the nearest centimetre (cm) using a wall‐mounted rule. BMI was calculated as mass (kg) divided by height in metres squared (kg/m^2^).^3^ United Kingdom Royal College of Paediatrics and Child Health World Health Organization growth charts were used to calculate and classify BMI‐centile in relation to age and sex.^28^ All measurements were taken by a single‐trained operator (HS) using the same equipment. Ethical approval was obtained from the United Kingdom National Research Ethics Service (UK NRES) (14/LO/0769). Written informed consent was received from all participants.

Bacterial DNA was extracted from saliva samples by means of the GenElute Bacterial Genomic DNA extraction kit (Sigma‐Aldrich) following manufacturer's instructions, with an additional lysozyme (45 mg/mL) incubation step for 30 minutes at 37°C. To assess the quantity of nucleic acid, the Qubit fluorometer was utilized to measure DNA (>10 picogram (pg)/mL detection limit) following manufacturer's instructions. In addition, agarose gel electrophoresis (1.5% agarose gel) was performed to assess the integrity of DNA.

For 16S rRNA gene sequencing, a previously published methodology was followed.^29^ Polymerase chain reaction (PCR) was performed using fusion primers combining the MiSeq adapter sequences (i5 and i7), an 8‐nt barcode sequence, a 10‐nt primer pad, a 2‐nt linker sequence and a 16S rRNA gene‐specific sequence targeting the V1‐V2 region of the 16S rRNA genes of bacteria, with near universal specificity. The primer sequences were as follows: Forward PCR primer ‐ 27F‐YM 5′‐ AATGATACGGCGACCACCGAGATCTACACXXXXXXXXAGTCAGTCTGTCAGAGTTTGATYMTGGCTCAG‐ 3′; Reverse PCR primer ‐ 338R‐R 5′‐ CAAGCAGAAGACGGCATACGAGATXXXXXXXXTATGGTAATTCATGCTGCCTCCCGTAGRAGT ‐3′. Barcode combinations in the forward and reverse primers allowed dual indexing of the amplicons. PCR was performed in 25 microlitre (μl) reaction volumes in sterile 96‐well PCR plates (Thermo‐Scientific) with 12.5 μl of Extensor Hi‐Fidelity PCR Reddymix master mix (Thermo‐Scientific), 9.5 μl of sterile molecular biology‐grade water (Sigma), 0.5 μl of forward primer (at 10 micromolar, μmol/L), 0.5 μl of reverse primer (at 10 µmol/L) and 2 μl of DNA template. PCR conditions were 5 mins at 95°C followed by 25 cycles of 95°C for 45 seconds, 53°C or 45 seconds, 72°C for 45 seconds and a final extension of 72°C for 5 minutes. Amplicons were purified and their concentration normalized using the SequalPrep plate normalization kit (Invitrogen). Sequencing was performed by the King's College London Genome Centre using the Illumina MiSeq instrument and standard protocols for 2x250 basepair (bp) paired‐end sequencing.

### 2.5 Data analysis

Sequences were analysed using the mothur analysis pipeline (version 1.40.5)^30^ following the MiSeq SOP. DNA sequences were excluded if they were longer than 350 bp with homopolymers of greater than 8 bp. The Human Oral Microbiome Database reference dataset (version 13) was used to classify sequences after quality filtering and chimera removal. Sequences were grouped into Operational Taxonomic Units (OTU) using a sequence identity threshold of 98.5%. The alpha diversity of the subject groups was compared as the observed number of OTUs (richness) and the Inverse Simpson statistic (diversity). Sample composition across patient groups was displayed by means of a heatmap. Analysis of Molecular Variance (AMOVA) was performed to determine if there were significant differences in bacterial community composition between patient groups. Principal Coordinate Analysis (PCoA) was performed on distance matrices, generated with the Jaccard Index and thetaYC metric and plots prepared showing β‐diversity comparisons.

## 3 RESULTS

### 3.1 Participants

Saliva was obtained from 19 adolescent patients with an overall mean (SD) age 15.6 (1.8) years. These patients were divided into 10 normal‐weight with mean BMI of 21.6 (3.5) kg/m^2^ and 9 obese with a mean BMI of 30.7 (3.1) kg/m^2^. Both groups had healthy gingival tissues with comparable gingival and plaque indices. Baseline demographic data are shown in Table 1.

**TABLE 1:** Patient demographics at baseline

	Overall	Normal‐weight	Obese
Patients (n)	19	10	9
Male/female (n)	11/8	4/6	7/2
Age	15.6 (1.8)	15.2 (1.6)	16 (1.9)
BMI kg/m^2^	25.4 (5.6)	21.6 (3.5)	30.7 (3.1)
uWMS ml/mm	0.58 (0.22)	0.59 (0.28)	0.53 (0.26)
Plaque index	0.46 (0.22)	0.50 (0.25)	0.42 (0.20)
Gingival index	0.59 (0.32)	0.58 (0.27)	0.60 (0.38)

Note

For demographics: values are mean (SD) unless otherwise indicated.

### 3.2 PCR and bacterial sequencing data

A total of 156 783 sequences were obtained from the samples after filtering for length and quality, with 4678 sequences sub‐sampled from each sample to normalize the data. The richness and diversity of the sample by patient group is shown in Table 2. There were no significant differences in richness or diversity of the samples (Wilcoxon 2‐sample ranked test).

**TABLE 2:** Richness (mean number of OTUs) and diversity (Inverse Simpson index) of samples by obesity status and gender

Patient group	n	Mean number of OTUs (SD)	Inverse Simpson index (SD)
Normal	10	334.4 (50.2)	12.9 (6.0)
Obese	9	393.4 (78.8)	20.3 (9.9)
Male	11	366.2 (80.2)	17.6 (8.8)
Female	8	357.0 (58.0)	13.8 (9.2)

**FIGURE 1:**
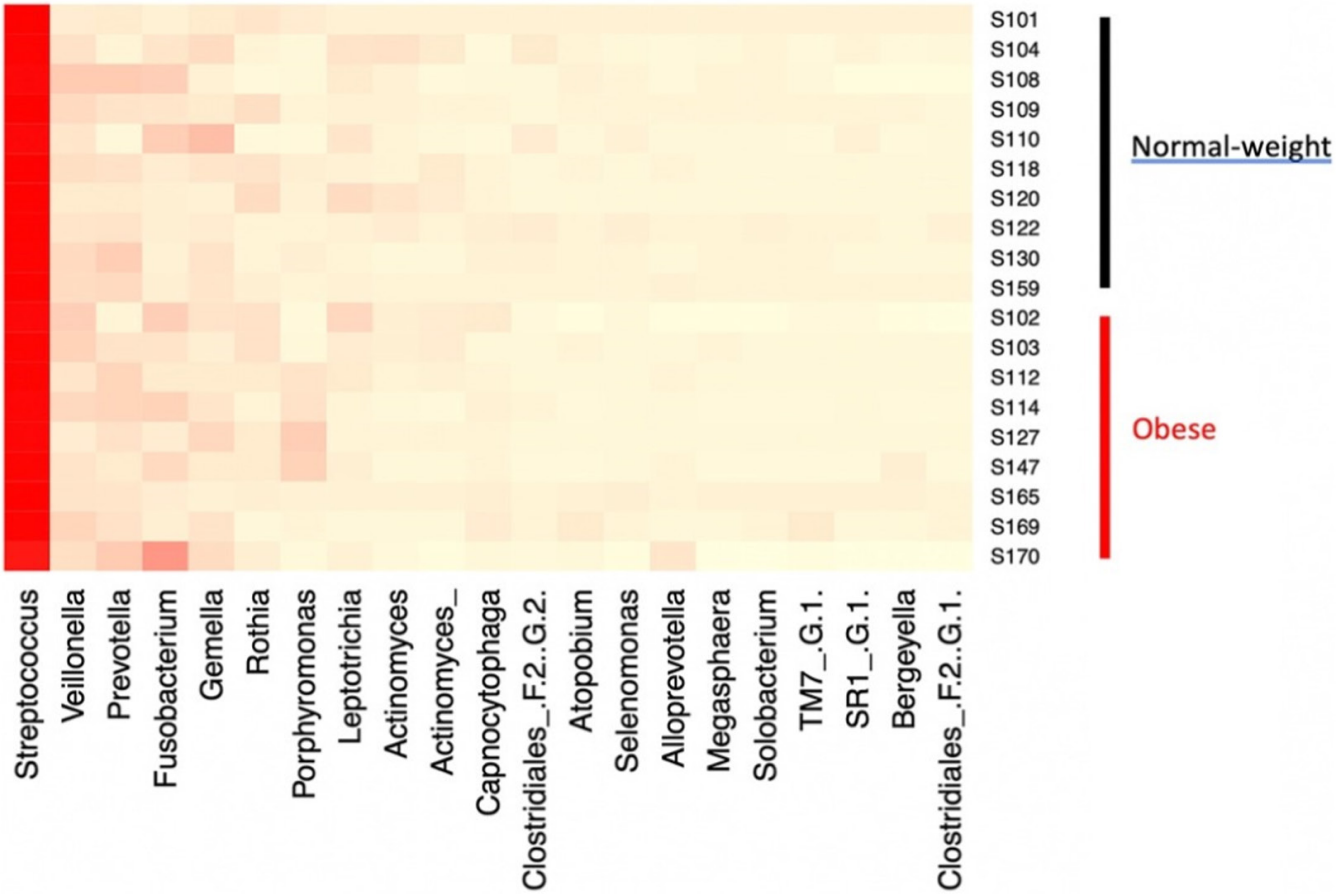
Heatmap showing predominant bacterial genera amongst patients by BMI

**FIGURE 2:**
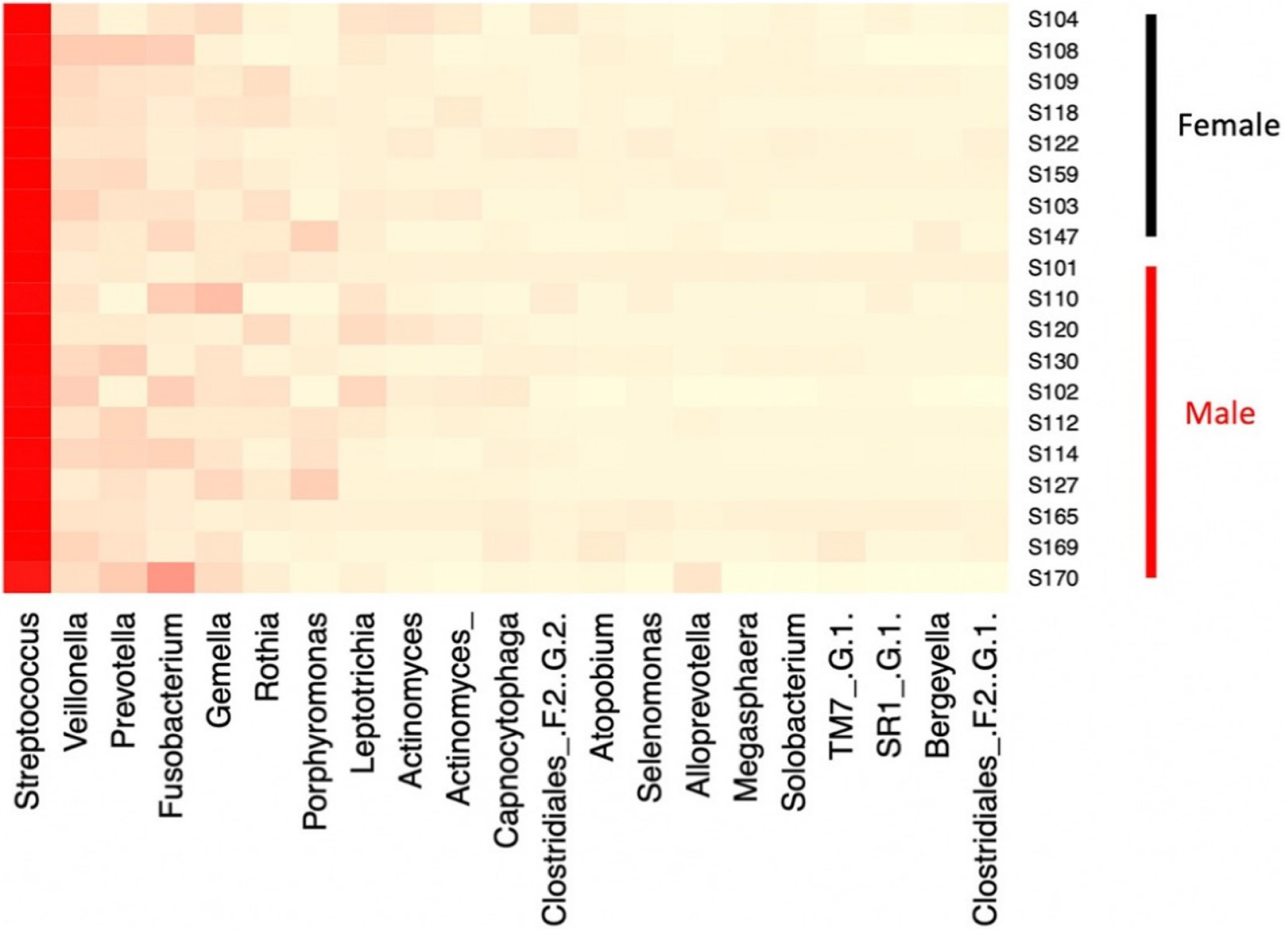
Heatmap showing predominant bacterial genera amongst patients by gender

**FIGURE 3:**
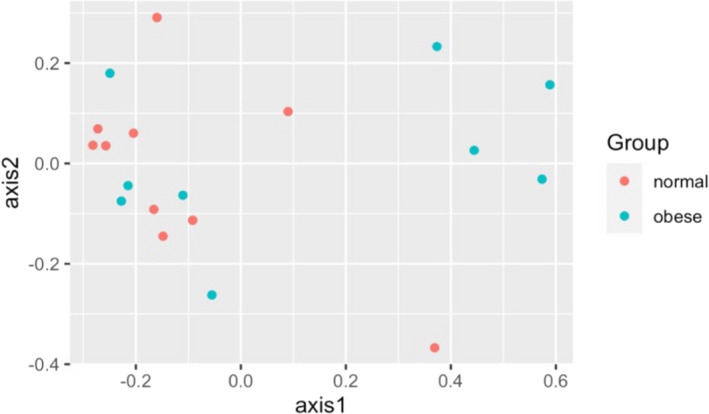
Principal Coordinate Analysis plot comparing bacterial community structure of patient groups by BMI

**FIGURE 4:**
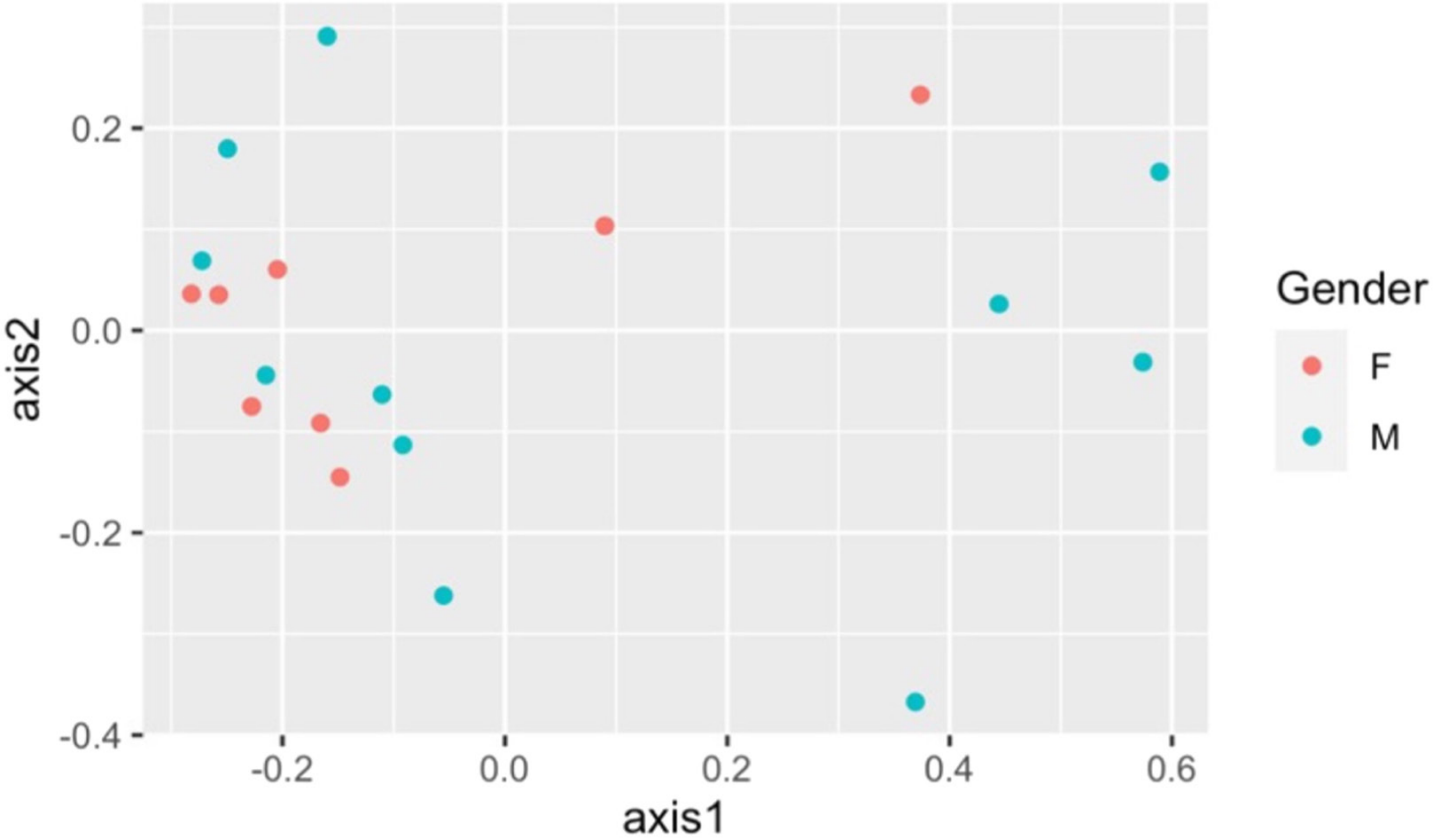
Principal Coordinate Analysis plot comparing salivary bacterial community structure by gender

The composition of bacterial communities in the different patient groups is shown in Figures 1 and 2. The samples were dominated by bacterial genera characteristic of the human oral cavity including S*treptococcus*, *Porphyromonas*, *Veillonella*, *Gemella*, *Prevotella*, *Fusobacterium* and *Rothia*. There were no significant differences in the alpha or beta diversity of bacterial communities in the mouth of normal‐weight and obese patients and no gender‐related differences (AMOVA). This was confirmed visually with scattering of the data in the PCoA plot, a method to visualize similarities and dissimilarities across data (Figures 3 and 4). Obese adolescents attending for orthodontic treatment had a similar microflora to their normal‐weight counterparts.

## 4 DISCUSSION

Obesity correlates with a number of chronic diseases and inflammatory disorders.^2^ The pro‐inflammatory activity of adipose tissue can contribute to reduced body immunity and increased susceptibility to infection.^31^ The presence of obesity has also been associated with increased risk of dental disease^4^, ^5^ with periodontal disease severity positively correlated with increased BMI.^32^ Moreover, a recent prospective study revealed significant differences in baseline GCF levels of several pro‐inflammatory adipokines and inflammatory mediators in obese subjects compared to normal‐weight, suggesting an altered inflammatory state in the periodontal tissues of matched obese adolescents.^3^

Orthodontic appliances carry the risk of periodontal tissue damage and enamel decalcification when optimal oral hygiene is not present and maintained.^21^ A recent systematic review investigated the effect of removable orthodontic appliances on oral microbiota and showed significant alterations in oral pathogens with increases in *Candida*, *Lactobacillus*, *Moraxella catarrhalis* and *Staphylococcus epidermidis* in the first months of therapy, *Streptococcus mutans* in the first 15 days and subgingival *Spirochaetes* significantly increased during the 6‐7 months of treatment. These microbial changes returned to pre‐treatment level few months after the completion of treatment.^33^ Whether orthodontic treatment in obese adolescents increases this risk has not been investigated. We therefore explored the salivary bacterial community in obese and normal‐weight cohorts to investigate potential salivary dysbiosis in obese adolescents that might place them at increased risk of dental disease relevant to orthodontic treatment with fixed appliances.

Current evidence suggesting a difference between the salivary microbiome amongst obese and normal‐weight patients is unclear. It has been reported that obesity can impact on the salivary microbiome in adolescents, with divergence in the bacterial community related to both sex and body mass.^34^ Specifically, there was an increase in the abundance of *Rothia* and *Neisseria* amongst obese male and female groups, whilst obese females showed an abundance of *Rothia* compared to normal‐weight. In addition, *Haemophilus* spp. was higher amongst normal‐weight females. In male adolescents, the obese group presented with higher *Neisseria* spp. compared to *Veillonella* spp., whilst *Prevotella* spp. was dominant in the normal‐weight.^33^ Similar studies have reported the association of salivary microbial diversity and composition with BMI being gender‐related.^35^, ^36^ In a Finnish study of underweight, normal, overweight and obese children (of which 75% were normal‐weight), the majority of samples were composed of the core saliva microbiota *Veillonella, Prevotella, Streptococcus, Neisseria, Selenomonas, Haemophilus, Eubacterium, Porphyromonas, Fusobacterium, Gemella, Campylobacter, Granulicatella, Leptotrichia*, and *Johnsonella*,^36^ which are consistent with our present study. Although there were alpha and beta diversity differences between obese and normal‐weight adolescents in the Finnish study, with reduction in the core bacteria: *Veillonella, Prevotella, Streptococcus, Selenomonas*, and *Neisseria* amongst the obese participants, these differences were gender‐related, suggesting the influence of genetic and physiological factors. Overall, it was concluded that no statistical differences existed between obese and normal‐weight cohorts.^36^

In the present study, there were no overall significant differences in richness or diversity of the samples by obesity status or gender. All groups were dominated by bacterial genera characteristic of the human mouth, particularly *Streptococcus*, *Porphyromonas*, *Veillonella*, *Gemella*, *Prevotella*, *Fusobacterium* and *Rothia*. This is consistent with other previous studies.^9^, ^11^, ^17^ Stahringer and colleagues recruited 107 participants aged 8 to 26 and utilized saliva samples to find no association between gender and BMI with any change in saliva microbiome profile at the taxonomic level.^17^ Similarly, no correlation between BMI and change in salivary bacterial diversity was found in a sample of 25 females, divided into those with polycystic ovary syndrome and healthy groups.^13^ Moreover, a more recent study reported a lack of diversity at the species level amongst subjects with Type 2 diabetes, obese and normal‐weight adolescents using alpha diversity, although they attributed the lack of correlation to the small sample size in each group.^9^

In terms of the influence of orthodontic treatment on the salivary microbiome, a prospective study investigated the effect of fixed and removable orthodontic appliances amongst adolescent patients on salivary bacterial community. They reported significant changes in salivary microbiota composition with increases in *S. mutans*, lactobacilli and *Candida albicans*.^37^ In addition, the salivary microbiome of 23 subjects undergoing orthodontic treatment showed a significant increase in *S. mutans* and lactobacilli after insertion of the orthodontic appliances.^38^ Therefore, orthodontic appliances create a new environment with shift towards species associated with caries such as *S. mutans* and lactobacilli as well as periodontitis *P. gingivalis, T. forsythia* and *T. denticola*.^22^ Whilst alteration in the oral microbiome was reported with significant increase in streptococci, there was no statistical elevation in periodontitis‐associated bacteria from pre‐treatment to 1 year.^20^ A recent randomized clinical study of 120 adolescent subjects who received orthodontic treatment with or without fluoride mouthwash showed that during orthodontic treatment, there was rise in *Selenomonas* and *Porphyromonas* counts, whilst *Streptococcus*, *Rothia* and *Haemophilus* were abundant towards the end of orthodontic treatment. They concluded that as long as oral hygiene is maintained, orthodontic treatment does not adversely affect oral health.^23^ Our study analyses confirmed lack of statistical difference in the salivary bacterial environment at baseline amongst obese adolescents and their normal lean at the baseline (T1) before insertion of orthodontic appliances, thus interestingly suggesting that obese adolescents are not at increased risk of adverse oral health when considering orthodontic fixed appliances.

The main strength of the present study was the exclusion of factors that could potentially influence the saliva microbiome, such as the exclusion of those classified as underweight (BMI‐centile <2) and overweight (BMI‐centile 91‐98), smokers, recent history of antibiotic usage and periodontal disease. In addition, oral health parameters such as gingival and plaque indices were assessed and were comparable in both cohorts at baseline. Moreover, the study had a prospective design.^39^ However, one of the potential limitations was the sample size calculation, which was not based specifically on the salivary microbiota but powered based on the primary outcome of the original study, which was time to alignment completion with fixed appliances.^3^ Our sample size in each group was relatively small, and this could have affected the results of lack of association amongst the cohorts. Another limitation was using only BMI to classify adiposity, which might have limited the identification of overweight participants and could have been overcome by using other indices such as fat mass index and fat distribution.^3^, ^40^ However, to date there has been no study investigating the effect of obesity on the salivary microbiome amongst adolescent population undergoing orthodontic treatment with fixed appliances.

In summary, although our study did show an increase in richness of the bacterial community at the level of OTUs amongst obese adolescent subjects compared to normal‐weight prior to placement of fixed appliances, this was not statistically significant. In addition, there were no gender‐related differences in the salivary bacterial community of these groups, suggesting that the intra‐oral microbiological profile of obese adolescents does not make them at increased risk of periodontal disease during orthodontic treatment.

## CONFLICT OF INTEREST

The authors declare no potential conflicts of interest with respect to the authorship and/or publication of this research article.

## AUTHOR CONTRIBUTIONS

MC, GC, HS and SS conceived the project; SS and LC undertook data extraction; SS, LC, MC and GC interpreted the results and wrote the manuscript.

## Data Availability

The data that support the findings of this study are available from the corresponding author upon reasonable request.
